# A Combination of Serological Assays to Detect Human Antibodies to the Avian Influenza A H7N9 Virus

**DOI:** 10.1371/journal.pone.0095612

**Published:** 2014-04-22

**Authors:** Libo Dong, Hong Bo, Tian Bai, Rongbao Gao, Jie Dong, Ye Zhang, Junfeng Guo, Shumei Zou, Jianfang Zhou, Yun Zhu, Li Xin, Xiaodan Li, Cuiling Xu, Dayan Wang, Yuelong Shu

**Affiliations:** National Institute for Viral Disease Control and Prevention, China CDC, Key Laboratory for Medical Virology, National Health and Family Planning Commission, Beijing, China; Center for Biologics Evaluation and Research, United States of America

## Abstract

Human infection with avian influenza A H7N9 virus was first identified in March 2013 and represents an ongoing threat to public health. There is a need to optimize serological methods for this new influenza virus. Here, we compared the sensitivity and specificity of the hemagglutinin inhibition (HI), microneutralization (MN), and Western blot (WB) assays for the detection of human antibodies against avian influenza A (H7N9) virus. HI with horse erythrocytes (hRBCs) and a modified MN assay possessed greater sensitivity than turkey erythrocytes and the standard MN assay, respectively. Using these assays, 80% of tested sera from confirmed H7N9 cases developed detectable antibody to H7N9 after 21 days. To balance sensitivity and specificity, we found serum titers of ≥20 (MN) or 160 (HI) samples were most effective in determining seropositive to H7N9 virus. Single serum with HI titers of 20–80 or MN titer of 10 could be validated by each other or WB assay. Unlike serum collected from adult or elderly populations, the antibody response in children with mild disease was low or undetectable. These combinations of assays will be useful in case diagnosis and serologic investigation of human cases.

## Introduction

In March 2013, the first documented infection of humans with a novel avian influenza A (H7N9) virus was identified in China [1]. After the first wave of 133 cases from February to May 2013, only 2 cases were found in June and July, while the second wave had been occurring since October 2013. As of February 21^st^, 2014, 356 of H7N9 infection were have been reported in mainland China with 114 death and 5 cases in Hong Kong and 2 cases in Taiwan [2]. The dual receptor-binding profile of H7N9 and the limited detection of human-to-human transmission highlight the pandemic potential of this virus [3], [4]. Similar to human infection with H5N1 viruses, most human cases typically present with severe pneumonia [5], and only few mild cases with fever have been reported [6].

Serological assays serve a critical role in the identification of mild and asymptomatic infections caused by H7N9 viruses in humans. Previous studies have developed a combination of serological assays, including the microneutralization (MN) assay, hemagglutinin inhibition (HI), enzyme-linked immunosorbent assay (ELISA), and Western blotting (WB) to detect human anti-H5N1 antibody [7] ^,^ [8]. However, compared with H5N1 virus infection, numerous studies using the classic serum antibody assays of HI and MN have demonstrated that H7 subtype virus infection typically induces low titers of anti-H7 antibodies [9]. Thus, there remains a need to comprehensively evaluate the sensitivity and specificity of different serological assays to detect human antibodies against H7 viruses in general, and H7N9 viruses in particular.

To improve existing serologic assays to detect H7N9-specific antibody, we optimized the HI assay with RBCs from different species, modified the MN assay with different test parameters, and developed the WB assay to improve the antibody detection of H7N9 infection. The sensitivity and specificity of the HI and MN assays were evaluated using 15 serum samples of convalescent sera of H7N9 patients and 258 control serum samples. The kinetics of the human antibody response in young, adult, and elderly populations was analyzed based on the assays developed in this study.

## Materials and Methods

### Serum Samples

Forty-seven serum samples were collected from 36 patients with H7N9 infection (age range, 3.8–87 years) between April 2^nd^ and June 28^th^, 2013 (Table S1). The presence of viral infection was confirmed by real-time reverse-transcriptase polymerase chain reaction (PCR) and/or virus isolation (Table S1). Thirty-two serum samples were collected within 14 days of illness onset; the remaining 15 serum samples were obtained after 21 days (Table 1). Sera collected <7 days after illness onset were referred to as acute-phase samples. Serum samples collected 7–13 days were considered to likely contain H7-specific antibody, while those that had been collected ≥14 days after illness onset were referred to as convalescent-phase samples.

**Table 1:** The information of test serum samples.

	H7N9 patient group (N = 47)					Non-H7N9 patient group (N = 258)			
Description	Single serum sample	Paired serum samples (8 persons)						Poultry workers	
	Collected<14 daysafter illness	Collected >21days afterillness	Collected<14 daysafter illness		Collected >21days afterillness	Sera of General population	Sera with fivetypes of seasonalinfluenza antibodies	With H5N1antibody	With H9N2antibody
Test antigens	H7N9					H1N1 2009 pdm	5 seasonal influenza virus	H5N1	H9N2
No.of serum samples	21	7		11	8	94	100	15	49
Average age (range)	57 (3.8–87)	53 (30–75)		35 (4–69)		21 (18–39)	24 (1–79)	36 (8.9–70)	44.6 (26–70)
Antibody GMT of HI titer (range)	12 (5–160)	430 (160–1280)		8 (5–80)	54 (5–640)	15 (5–128)	65–130 (40–2560)	105 (80–640)	(80–>160)
Testing methods	HI, MN,WB					HI, MN	HI, MN,WB*	HI, MN,WB**	HI, MN,WB**

Abbreviations: HI, hemagglutination inhibition assay; MN, microneutralization assay; WB, Western blot assay; GMT: geometric mean titers. WB*, eight serum samples were tested with the WB assay. WB**, one serum sample was used in the WB assay. Titers below 10 were considered negative and assigned a value of 5. 5 seasonal influenza viruses: H1N1, H3N2, H1N1 2009 pdm, B Victoria, B Yamagata.

A total of 258 control serum samples collected from 2010–11 were obtained from subjects (age range, 1–79 years) for specificity testing as follows: 94 from the general population in Shanghai collected in January 2010, 100 from individuals in Mongolia collected in June 2010 that simultaneously contained HI antibodies against five seasonal influenza viruses (H1N1 [40–640], H3N2 [40–640], H1N12009 pandemic [40–2560], B Victoria [40–640], and B Yamagata [40–640]) (Table 1); 15 sera obtained from poultry workers and patients in China with antibody titers (80–640) against H5N1 virus; and 49 from poultry workers in China with antibodies to H9N2 virus (80–160) collected in 2011. All control sera were stored at –20°C until testing. This study was performed according to the National Pandemic Preparedness and Response Plan; therefore, the waiver exempting of the subject institution review was approved by the human research ethics Committee of National Institute for Viral Disease Control and Prevention, Chinese Centers for Disease Control and Prevention. The data used in our study was anonymous prior to analysis.

### Virus, RBCs and Animal Sera

Influenza A/Anhui/1/2013 was used as a representative H7N9 virus in all HI and MN assays. All experiments with live H7N9 viruses were conducted in a biosafety level 3 laboratory. RBCs were derived from turkeys, horses, goose and rabbits held at the Fangyuan experimental animal farm, Beijing. Ferret antiserum against A/Anhui/1/2013 and rabbit antiserum against A/Environment/Dongting/PC360/2011 (H7N7) isolated from Dongting lake were used as positive controls in both assays. Ferret antibodies against the human seasonal influenza viruses pandemic H1N1 (A/California/7/2009) and H3N2 (A/Victoria/361/2011) were used as negative control sera. The RBCs and animal sera raised against influenza protocols were approved by the Animal Care Welfare Committee of National Institute for Viral Disease Control and Prevention, Chinese Centers for Disease Control and Prevention.

### HI and MN Assays

For the HI assay, all sera were inactivated at 56°C for 30 min and adsorbed with hRBCs or turkey RBCs (tRBCs) for 30 min at 4°C to remove nonspecific hemagglutinin. Following absorption, the sera were treated with receptor-destroying enzyme (RDE; Denka-Seiken, Japan) at 37°C overnight and then tested using hRBC HI [10] and tRBC HI [11]. For the standard MN assay, two fold serial dilutions of 50 µL sera were mixed with an equal volume of influenza virus at 2×10^3^ TCID_50_/mL in diluents containing 1% bovine serum albumin (BSA) and plated in 96-well immunoassay plates. After incubation for 1 h at 37°C, 100 µL of 1.5×10^5^ cells/mL Madin-Darby canine kidney cells (MDCK) was added to each well as described previously [7]. To improve the sensitivity of the MN assay, the cell concentration, BSA concentration, and virus titer were optimized. MDCK cells were used at concentrations of 1.5×10^5^, 1.0×10^5^, and0.5×10^5^ cells/mL, BSA was used in assay diluents at concentrations of 1.0, 0.5, and 0%, and virus was used at concentrations of 2×10^3^and 1×10^3^ TCID_50_/mL. The final optimization parameters of MN assay (modified MN) were 1.0×10^5^ cells/mL cells, 0.5% of BSA and 50 ul of 1×10^3^ TCID_50_/mL virus. For both assays, serial two-fold dilutions (1∶10 to 1∶1280) of serum were used at a starting dilution of 1∶10, with titers expressed as the reciprocal of the highest dilution of complete hemagglutination or 50% neutralization. Titers below 10 were considered negative and assigned a value of 5. HI and MN titers were transformed to log2 for the statistical analyses. 95% confidence intervals for sensitivity and specificity and the t-test were calculated using OpenEpi, Version 2.

### WB Assay

The WB assay was adopted from Rowe et al. with several modifications [7]. The baculovirus-expressed recombinant HA protein (58 kDa, 517 amino acids; Sino Biological, Inc.) of the A/Anhui/1/2013 (H7N9) virus was used for the WB assay. HA protein was loaded onto each lane for standard SDS-PAGE running and membrane transfer, with membranes blotted overnight at 4°C. Diluted human sera were added to each lane and incubated at room temperature for 1hour. Anti-HA antibodies of A/Anhui/1/2013(H7N9) virus rabbit sera (Sino Biological, Inc.) were used as the positive control. Five human sera containing pandemic H1N1, seasonal H1, seasonal H3, H5N1, and H9N2 antibodies respectively were used as negative controls (Table 1). Following incubation with the secondary horseradish peroxidase (HRP)-conjugated goat anti-human and anti-rabbit IgG (KPL Inc.), blots were developed with tetramethylbenzidine (TMB) stabilized substrate (Promega Corporation) modified from previous descriptions [7].

## Results

### HI and MN Optimization

**Figure 1:**
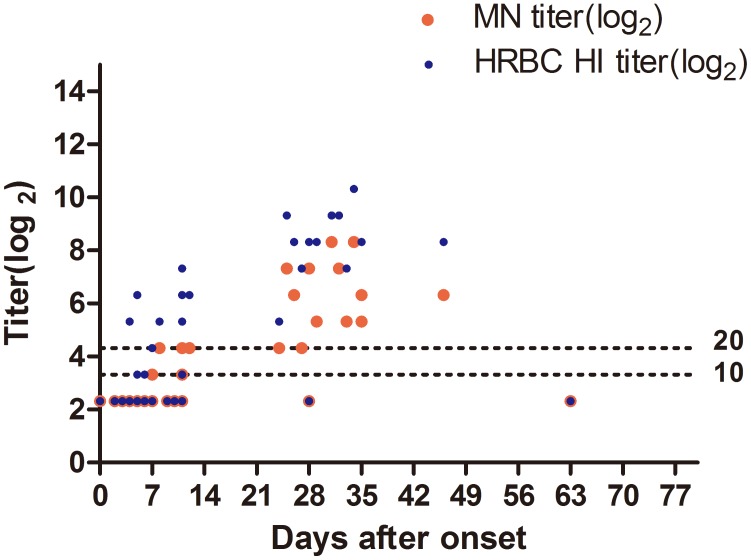
Spectrum of antibodies against influenza A H7N9 virus by along the days after illness onset. Forty-seven serum samples were collected from 36 patients with H7N9 infection between April 2nd and June 28th were tested by both horse erythorocytes hemagglutinin inhibition (hRBC HI), the modified microneutralization (MN) to detect H7-specific antibody.

Similar to lower antibody responses detected in previous H7 subtype infection in humans [9], the first two available convalescent sera from H7N9 infected individuals only achieved titers of 40 using tRBC HI and titers of 20 and 40 by MN. To improve the H7N9 antibody detection rate, numerous parameters of each serologic assay were modified and examined for heightened sensitivity and specificity against human serum samples collected from H7N9 virus-infected patients. Of 47serum samples available for testing, we identified 20 that had both HI and MN titers and were concurrently positive by WB assay (Figure 1, Figure S1), and were chosen for further analysis in these modified assays.

Horse erythrocytes (hRBCs) were found to be more sensitive than turkey erythrocytes (tRBCs) for the detection of avian H5N1 influenza antibodies in mammals via the hemagglutination inhibition (HI) assay, but it was unknown if H7N9 viruses would display a similar profile. Erythrocytes from four mammalian species (horse, turkey, goose, and rabbit) were evaluated in HI assays against the novel virus strain. As compared with tRBCs, the use of hRBCs increased the sensitivity of the HI assay from 74% to 95%, 58% to 90%, 32% to 75%, and 11% to 65% at cutoff titers of ≥20, ≥40, ≥80, and ≥160, respectively. Goose and rabbit RBCs provided unstable results and are not included here (data not shown).

To optimize the MN assay, varying concentrations of virus, cells, and diluents were examined for the ability to achieve higher H7N9 virus and sera titers. A modified MN using 1.0×10^5^cells/mL cells, 0.5% of BSA and 50 ul of 1×10^3^ TCID_50_/mL virus was selected as the final optimization condition. This modified assay increased the sensitivity of the H7N9 antibody detection rate from 70% to 100%, 65% to 85%, 45% to 55%, 5% to 40%, and 2% to 25% at cutoff titers of ≥10, ≥20, ≥40, ≥80 and ≥160 respectively (Table 2). Based on these results, the hRBC HI and modified MN assays were used in the further sensitivity and specificity testing.

**Table 2:** The comparation of sensitivity of standard MN and Modified MN, tRBC HI and hRBC HI with sera of H7N9 cases.

Testing Titers	Sensitivity % (95% CI)*
**TRBC HI**	
HI≥20	74 (51–90)
HI≥40	58 (35–78)
HI≥80	32 (14–55)
HI≥160	11 (2–31)
**HRBC HI**	
HI≥20	95 (78–100)
HI≥40	90 71–98)
HI≥80	75 (53–90)
HI≥160	65 (43–83)
**Standard MN**	
MN≥10	70 (48–87)
MN≥20	65 (43–83)
MN≥40	45 (25–67)
MN≥80	5 (10–47)
MN≥160	2 (2–29)
**Modified MN**	
MN≥10	100 (86–100)
MN≥20	85 (64–95)
MN≥40	55 (33–75)
MN≥80	40 (21–62)
MN≥160	25 (10–47)

Note: *, Using 20 sera with MN, HI titer and WB band (collected during 7–63 days).

95% CI was calculated using OpenEpi, Proportion using Mid-P Exact test.

### WB Assay Development and Optimization

WB assays have been used for diagnosis of H5N1 infection in humans, but have not been widely employed in serologic studies with H7 subtype-exposed individuals. In order to optimize the WB assay for detection of H7N9 antibody, human sera containing anti-H1N1 2009 pdm (tRBC HI titer, 640), anti-seasonal H1N1 (tRBC HI titer, 320), anti-seasonal H3N2 (guinea pig RBC HI titer, 80), anti-H5N1 (hRBC HI titer, 640), and anti-H9N2 (tRBC HI titer,160) were used as the negative controls. Rabbit serum against the recombinant HA protein of A/Anhui/1/2013(H7N9) virus and a serum sample from an H7N9 case (MN titer, 80) were used as positive controls. Recombinant HA protein (1000, 500, 250, and 125 ng), serially diluted human sera (1∶500, 1∶1000, and 1∶2000 dilutions), and serially diluted secondary antibodies (1∶1000 and 1∶2000 dilutions) were evaluated in the WB assay. The use of 250 ng of HA protein, a 1∶2000 dilution of human sera, and 1∶2000 dilution of the secondary antibodies yielded no cross-reactivity with negative human control sera, was used for further analysis of H7N9 human sera (Table 1, Figure S1).

### Correlation of HI, MN and WB Assays

Once the HI, MN, and WB assays had been optimized, 47 sera from confirmed cases were tested by all assays for the presence of antibody to H7N9 influenza virus (Fig. 1, Figure S1). A strong positive correlation (Pearson, r = 0.93, P<0.05) was noted between hRBC HI and MN titers. The hRBC HI titer was generally 4 fold higher than the MN titer. All sera with a MN titer of ≥10 (range of 10–320) were positive by WB, and sera samples with an HI titer of ≥160 were confirmed by MN and WB. 2 of 8 sera collected <7 days after illness with HI titer of 40–80 were negative by MN and WB assays. This acute serum samples results indicated that the HI titer of 80 may be negative when tested by the other assay and should be checked again for conformation.

### Sensitivity and Specificity of Serology Tests

The sensitivity (using 15 sera from cases collected ≥21 days after illness onset) and specificity (using 258 non-H7N9 infected human sera) of the antibody detection assays were tested with hRBC HI and MN assays (Table 3). When cutoff titers of ≥80 and 160 (HI) and ≥10 and 20 (MN) were used, the sensitivity reached 80% and 87% for each assay, respectively, with 100% specificity for both assays, indicating they would be suitable for use as cutoffs in single sera tests. Use of an HI cutoff titer of 20–40 increased the sensitivity to 87% and reduced the specificity to 97–98% (Table 3). Combining the HI titer of 20–40 with the MN or WB assay increased the specificity of the HI assay to 100%. MN titers of 10 and 20 exhibited comparable sensitivity (87%) and specificity (100%Table 3). While a titer of 10 was the minimum detection limit of the assay, to get the stable results for the replication, the MN cutoff titer of 20 of single serum will be suggested for sera positive. As all 15 convalescence sera at least of HI titer of 160 and one acute sera of HI titer 80 were negative for the MN and WB assays, thus the HI cutoff titer will be suggested as 160. Considering that sera collected in the earliest stages of illness or from mild cases may possess the lowest antibody titers, the WB assay could be used as a confirmatory test when sera displays an HI titer of 20–80 or MN titer of 10.

**Table 3:** The sensitivity and specificity of HI and MN assay for detection antibody to H7N9 virus.

	Sensitivity (%) (95% CI)	Specificity (%) (95% CI)
Sera number (average age)	15 (39.7 year)	258 (28.5 year)
**HRBC HI Assays**		
Cut off HI≥20	87 (62–98)	97 (94–98)
Cut off HI≥40	87 (62–98)	98 (96–99)
Cut off HI≥80	80 (55–95)	100
Cut off HI≥160	80 (55–95)	100
**Modified MN Assay**		
Cut off MN≥10	87 (62–98)	100
Cut off MN≥20	87 (62–98)	100
Cut off MN≥40	73 (47–91)	100
Cut off MN≥80	53 (29–77)	100
Cut off MN≥160	33 (13–59)	100

### Kinetics of Antibody Response Among Different H7N9 Case Age Groups

Of the 18 sera collected within 6 days after illness onset, none tested positive in the MN and WB assays, but two had hRBC HI titers of 10 and 40. Of the 14 sera collected between 7–14 days, 7 had HI titers of 10–160, MN titer of 10–20 and WB positive. Of the 15 sera collected after 21 days, 13 were positive in all the three assays (Figure1). The sero-conversion in six of the eight paired sera was detected by three assays.

As previous studies have identified that the magnitude of a serologic response to influenza virus infection can be influenced by the age of the patient, we next investigated the possible effect of age-specific sero-protection. The 47 human sera were divided by age into child, adult and elderly groups. The geometric mean titers (GMT) of the MN assay in the adult (GMT = 107.7, n = 7) and elderly (GMT = 69.6, n = 5) groups were higher than that in the child group (GMT = 7.9, n = 3) after 21 days after illness onset (Table 4). Convalescent sera of two out of the three children showed no detectable antibodies, indicating a low antibody response in children with mild symptoms.

**Table 4:** Analysis of antibodies to H7N9 by age group.

Age group	No. of cases	Average age (range)	Days after illness (range)		GMT of MN titer (range)	No. of serum samples with MN titer≥10 (%)	GMT of HI titer (range)	No. of sera with HI titer≥20 (%)
**Sera collection <7 days after illness onset**								
Child	4	6.8 (3.8–15)	2.2 (0–5)		5 (5)	0	5 (5)	0
Adult	4	41.3 (33–56)	4.8 (3–6)		5 (5)	0	8.4 (5–10)	3 (75)
Elderly	9	68 (60–81)	4.9 (4–6)		5 (5)	0	10 (5–80)	4 (44.4)
**Sera collection during 7–14 days after illness onset**								
Child	1	15 (15)	5 (5)		5 (5)	0 (0)	5 (5)	0 (0)
Adult	8	45.4 (33–55)	9.6 (7–12)		10.9 (5–20)	6 (75)	23.8 (5–160)	5 (62.5)
Elderly	5	74.6 (65–87)	8.6 (7–11)		6.6 (5–20)	1 (20)	8.7 (5–80)	1 (20)
**Sera collection >21 days after illness onset**								
Child	3	8.7 (4–15)	38.3 (24–63)		7.9^a^ (5–20)	1 (33.3)	10 (5–40)	1 (33.3)
Adult	7	43.0 (30–59)	30.9 (27–35)		107.7* (20–320)	7 (100)	431.7* (160–1280)	7 (100)
Elderly	5	69.2 (65–75)	33.0 (25–46)		69.6* (40–160)	5 (100)	320* (160–640)	5 (100)

Abbreviations: HI, hemagglutination inhibition assay; MN, microneutralization assay; GMT: geometric mean titers.

Note: *, P<0.05 comparing with the GMT of the child group a and b values, respectively, according to *t*-tests.

## Discussion

Due to the urgent need to establish serologic assays to identify individuals infected with a history of infection with H7N9 virus, especially individuals with mild or asymptomatic illness, we conducted an assessment of existing serologic assays to improve their sensitivity and specificity towards H7N9 antibody. The detection rate of H7N9 antibodies was improved by using hRBC HI instead of tRBC HI and by using modified MN instead of standard MN. According the sensitivity and specificity analysis, the cutoff titers of single sera for modified MN and hRBC HI assays of 20 and 160 were assigned, respectively.

While the use of horse erythrocytes has been frequently used for serologic testing of H5N1-suspected individuals, the unique dual receptor-binding propriety of the H7N9 virus necessitated further investigation regarding the most appropriate species of RBCs for the HI assay.[3]. The lower affinity binding of the avian virus to NeuGc-(2,3)Gal on hRBCs than on avian RBCs may explain the sensitivity of hRBCs [12]. H7 viruses associated with disease in humans display a range of receptor-binding properties; further study is needed to determine the most appropriate choice of erythrocytes for serologic assays for other viruses within this subtype.

The MN assay has been established as a critical assay in influenza serostudies, frequently demonstrating higher sensitivity to the HI assay. However, protocols for the use of MN in the diagnosis of avian influenza have been optimized for H5, and not H7, subtype viruses. We modified the traditional MN protocol by using fewer cells, lower BSA concentrations and reduced virus titers to achieve more sensitive results, while ensuring no cross-reactivity to H7N9 antibodies in the control sera. We suggested MN cutoff titer of 20 may not be adequate for a fourfold rise in seroconversion. However, the absence of antibody response in the control population guaranteed the high specificity of the MN assay when this cutoff was used in case diagnosis. While the titers of hRBC HI were usually higher than the modified MN titer, it will be reduced sensitivity with higher specificity overall using the MN assay.

Despite optimization, 13 of 15 convalescent sera in the confirmed cases were positive; the 2 negative samples belonged to the child group. A low antibody response in children was observed in H5N1 infection [7]. The serology of cases from Zhejiang showed that only 65.8% of the cases survived at antibody titers of ≥80 using recombinant H7N9 virus with tRBC [13]. It is clear that a more sensitive method needs to be established for testing the sera of children.

Only 60% of the cases showed low MN titers at 7–14 days after illness onset. Higher anti-H7N9 titers were detected after 21 days, indicating that the optimal collection time for sero-diagnosis is 3 weeks or more after illness onset. The antibody titers to H7N9 virus were low or none response in the child groups. Further studies are required to find the other immune protective response in cases of H7N9 in children.

A limitation of the sensitivity analyses is that only 15 convalescent sera were available for the study. The more serum sample available will provide more information about the immune response and sera diagnosis information of the H7N9 virus. As in all influenza sero-diagnosis, well-timed paired acute and convalescent sera that demonstrated a 4-fold or greater rise in antibody are the optimal approach for sero-diagnosis of H7N9 virus infection. Our results suggest that single sera with MN and HI titers of 20 and 160, respectively, should be considered H7N9-positive. Furthermore, sera possessing HI titers of 20–80 and MN titers of 10 could be confirmed by WB assay or each other. Considering the convenience of HI for screening large populations, our results indicate that serological investigation screening should be conducted with an hRBC HI titer of 20 followed by confirmation with MN or WB. The serological criteria for the H7N9 diagnosis may be adjusted according to different epidemiological stages of the novel H7N9 viruses in humans. The ELISA method of the serology is a promising method for testing influenza antibodies from children [7], it may be developed in the following study.

## Supporting Information

Figure S1 **Sera of H7N9 human patients tested by Western Blot assay.** 1∶2000 dilution of 47 human case sera and1∶2000 dilution of HRP-labeled anti-human IgG used as the secondary antibodies. M: pre-stained protein marker, 1–47: human case sera of H7N9, PC: anti-HA of H7N9 virus rabbit sera.
(TIF)

Table S1 **Characteristics of the cases of the sera used in serology assays.**
(DOCX)
