# Supplementary figures and images for: A Combination of Serological Assays to Detect Human Antibodies to the Avian Influenza A H7N9 Virus
Source: PLoS One. 2014 Apr 22;9(4):e95612. doi: 10.1371/journal.pone.0095612 (PMC3995704; doi:10.1371/journal.pone.0095612)

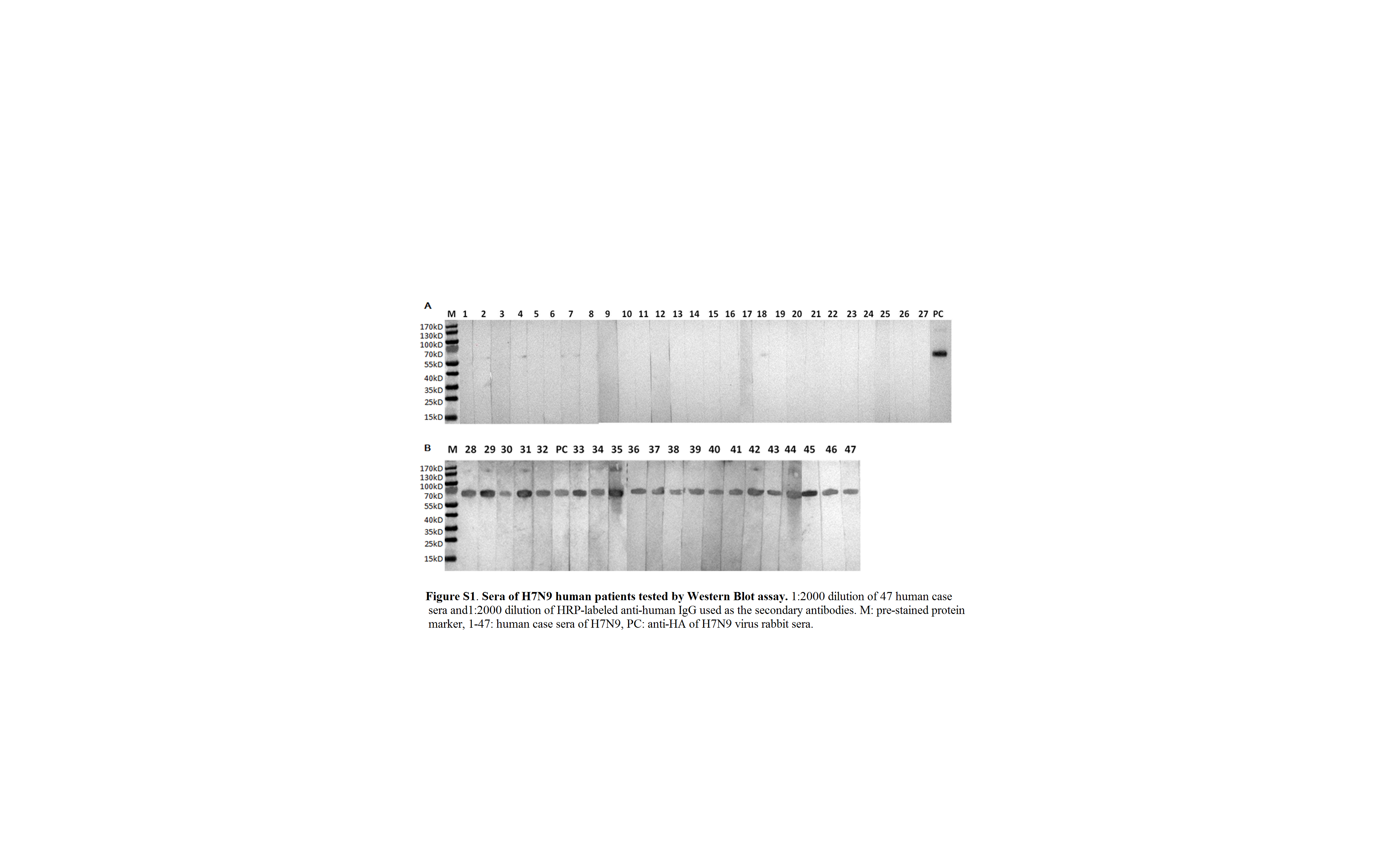

Supplement: Figure S1 — Sera of H7N9 human patients tested by Western Blot assay. 1∶2000 dilution of 47 human case sera and1∶2000 dilution of HRP-labeled anti-human IgG used as the secondary antibodies. M: pre-stained protein marker, 1–47: human case sera of H7N9, PC: anti-HA of H7N9 virus rabbit sera. (TIF) [file pone.0095612.s001.tif]
