# Supplementary material for: A Combination of Serological Assays to Detect Human Antibodies to the Avian Influenza A H7N9 Virus
Source: PLoS One. 2014 Apr 22;9(4):e95612. doi: 10.1371/journal.pone.0095612 (PMC3995704; doi:10.1371/journal.pone.0095612)
Supplement: Table S1 — Characteristics of the cases of the sera used in serology assays. (DOCX) [file pone.0095612.s002.docx]

Table S1. Characteristics of the cases of the sera used in serology assays.

| **No.** | **Description** | **Province** | **Gender** | **Age** | **Days after onset** | **Diagnosis Method** | **Outcome** |
| --- | --- | --- | --- | --- | --- | --- | --- |
| 1 | Single serum sample of acute-phase | Shanghai | M | 56 | 3 | Culture(+) | Recovered |
| 2 |  | Shanghai | M | 3.8 | 2 | Culture(+) | No record |
| 3 |  | Henan | M | 65 | 2 | RT-PCR(+) | Recovered |
| 4 |  | Shanghai | M | 68 | 6 | Culture(+) | Recovered |
| 5 |  | Shanghai | M | 87 | 7 | Culture(+) | Recovered |
| 6 |  | Jiangsu | F | 77 | 4 | Culture(+) | Dead |
| 7 |  | Anhui | M | 60 | 4 | Culture(+) | Recovered |
| 8 |  | Shanghai | F | 81 | 5 | Culture(+) | Recovered |
| 9 |  | Shanghai | M | 53 | 7 | Culture(+) | Recovered |
| 10 |  | Shanghai | M | 64 | 6 | Culture(+) | Dead |
| 11 |  | Shanghai | M | 63 | 6 | Culture(+) | Recovered |
| 12 |  | Henan | M | 24 | 7 | RT-PCR(+) | Recovered |
| 13 |  | Shanghai | F | 67 | 9 | RT-PCR(+) | Dead |
| 14 |  | Anhui | M | 55 | 7 | Culture(+) | Recovered |
| 15 |  | Jiangsu | M | 85 | 9 | RT-PCR(+) | Dead |
| 16 |  | Henan | M | 38 | 8 | Culture(+) | Recovered |
| 17 |  | Jiangsu | F | 33 | 11 | Culture(+) | Dead |
| 18 |  | Hunan | M | 54 | 10 | Culture(+) | Dead |
| 19 |  | Jiangsu | F | 45 | 11 | Culture(+) | Recovered |
| 20 |  | Hunan | F | 65 | 11 | Culture(+) | Dead |
| 21 |  | Jiangsu | F | 48 | 11 | RT-PCR(+) | Recovered |
| 22 | Single serum sample of convalescent-phase | Zhejiang | M | 43 | 32 | RT-PCR(+) | Recovered |
| 23 |  | Zhejiang | M | 55 | 34 | RT-PCR(+) | Recovered |
| 24 |  | Zhejiang | F | 68 | 33 | RT-PCR(+) | Recovered |
| 25 |  | Zhejiang | M | 75 | 35 | RT-PCR(+) | Recovered |
| 26 |  | Jiangxi | F | 30 | 29 | RT-PCR(+) | Recovered |
| 27 |  | Zhejiang | M | 59 | 31 | RT-PCR(+) | Recovered |
| 28 |  | Zhejiang | M | 48 | 28 | RT-PCR(+) | Recovered |

Table S1. Continued:

| **No.** | **Description** | **Province** | **Gender** | **Age** | **Days after onset** | **Diagnosis Method** | **Outcome** |
| --- | --- | --- | --- | --- | --- | --- | --- |
| 29-A | Paired serum samples | Fujian | M | 65 | 5 | Culture(+) |  |
| 29-C |  | Fujian | M | 65 | 25 |  |  |
| 30-A1 |  | Shandong | M | 4 | 0* | Culture(+) |  |
| 30-A2 |  | Shandong | M | 4 | 4 |  |  |
| 30-C |  | Shandong | M | 4 | 24 |  |  |
| 31-A1 |  | Shandong | M | 36 | 5 | Culture(+) |  |
| 31-A2 |  | Shandong | M | 36 | 12 |  |  |
| 31-C |  | Shandong | M | 36 | 35 |  |  |
| 32-A |  | Shanghai | M | 69 | 7 | RT-PCR(+) | Recovered |
| 32-C |  | Shanghai | M | 69 | 46 |  |  |
| 33-A |  | Shanghai | M | 40 | 5 | RT-PCR(+) |  |
| 33-C |  | Shanghai | M | 40 | 27 |  |  |
| 34-A |  | Hunan | M | 69 | 6 | Culture(+) |  |
| 34-C |  | Hunan | M | 69 | 26 |  |  |
| 35-A |  | Jiangsu | M | 15 | 0 | RT-PCR(+) |  |
| 35-A |  | Jiangsu | M | 15 | 11 |  |  |
| 35-C |  | Jiangsu | M | 15 | 63 |  |  |
| 36-A |  | Beijing | F | 7 | 5 | Culture(+) |  |
| 36-C |  | Beijing | F | 7 | 28 |  |  |

Table S1. Continued:

*：Serum sample was collected before onset of the patient in an active surveillance, the value of the days after onset is assigned as "0".
